# Rapid Regression of Carotid Artery Stenosis Shortly after Intensive Medical Therapy

**DOI:** 10.3390/tomography8010044

**Published:** 2022-02-21

**Authors:** Suh Yeon Park, Sang Hun Rhi, Ji Yeon Chung, Chan-Hyuk Lee, Byoung-Soo Shin, Hyun Goo Kang

**Affiliations:** 1 Jeonbuk National University Medical School, Jeonju 54907, Korea; qkrtjdus727@gmail.com (S.Y.P.); ymhb9627@gmail.com (S.H.R.); 2 Department of Neurology, Chosun University Medical School, Gwangju 61453, Korea; time4peace@hanmail.net; 3 Department of Neurology, Jeonbuk National University Hospital, Jeonju 54907, Korea; bluewave0210@gmail.com (C.-H.L.); sbsoo@jbnu.ac.kr (B.-S.S.); 4 Department of Neurology, Research Institute of Clinical Medicine of Jeonbuk National University-Biomedical Research Institute of Jeonbuk National University Hospital, Jeonju 54907, Korea

**Keywords:** carotid stenosis, atherosclerosis, hyperlipidemias, statin, aspirin

## Abstract

Carotid artery stenosis (CAS) is mainly caused by atherosclerosis. Intensive medical therapy is effective in preventing stroke in CAS. To date, there has been no published report of rapid regression of CAS. A woman with untreated hyperlipidemia visited our emergency room with left hemiparesis. She exhibited facial palsy, left hemiparesis, and dysarthria immediately after the visit. Brain magnetic resonance (MR) diffusion-weighted imaging confirmed acute infarction in the right middle cerebral artery (MCA) territory due to severe stenosis of the right internal carotid artery (ICA), which was revealed by MR angiography and carotid duplex ultrasonography. The patient started intensive statin therapy and dual antiplatelet agent therapy. Carotid artery stenting was not performed until hospitalization day 16 due to pleural effusion. On day 16, digital subtraction angiography was performed, and spontaneous regression of severe stenosis was observed. Only mild stenosis with ulcerative plaque was evident. The rapid CAS regression in this case may be caused by M2 macrophage polarization as a result of intensive statin therapy. This rapid regression may also result from reduced foam cell formation by statin and aspirin and thereby increased endogenous thrombolysis. Our patient demonstrated the efficacy of short-term intensive statin and aspirin therapy on atherosclerosis with untreated hyperlipidemia.

## 1. Introduction

Stroke is one of the major causes of death, with ischemic stroke accounting for 71% globally. Ischemic strokes are commonly caused by embolic sources occluding the cerebral artery, such as atherosclerotic plaques [1]. Foam cells, the most important contributor to atherosclerosis, are formed by lipids accumulating in the arterial wall, and atherosclerosis is caused by the accumulation of necrotized foam cells [2]. During the process, atherosclerotic plaque causes vascular stenosis as the diameter of the vascular lumen narrows or plaque ruptures to give rise to a thrombus on the site. Lowering low-density lipoprotein (LDL) to reduce foam cell formation is the current preventive treatment for atherosclerotic plaque and ischemic stroke.

Despite the large diameter of the carotid artery, occlusion accounts for 20–30% of strokes because the carotid bulb causes a complex blood flow pattern [3]. A patient who presents with ischemic events, including retinal artery occlusion, transient ischemic attack, and stroke, is diagnosed with symptomatic carotid artery stenosis (CAS). Symptomatic CAS accounts for 15% of ischemic stroke [3]. For symptomatic carotid artery stenosis greater than 50% of the vascular lumen, both surgical and medical treatment are indicated. Surgically, endarterectomy and carotid stenting may be performed. Medical therapy may include antiplatelet agents and statins [4]. According to Hankey et al., the recurrence rate of stroke is decreased by 12% with every 39 mg/dL reduction in LDL cholesterol and by 50% after 6–12 weeks of aspirin [5]. Therefore, intensive medical therapy is a highly effective treatment for secondary prevention of stroke in symptomatic CAS.

Recent studies have shown regression of carotid artery stenosis by intensive medical therapy, but the findings were related to long-term treatment [6,7]. To our knowledge, CAS regression by short-term intensive medical therapy has not been reported. Here, we report a rare case in which short-term intensive medical therapy resulted in rapid regression of symptomatic CAS with ischemic stroke before undergoing carotid artery stenting. We also conducted a literature review on the topic.

## 2. Case Presentation

**Figure 1:**
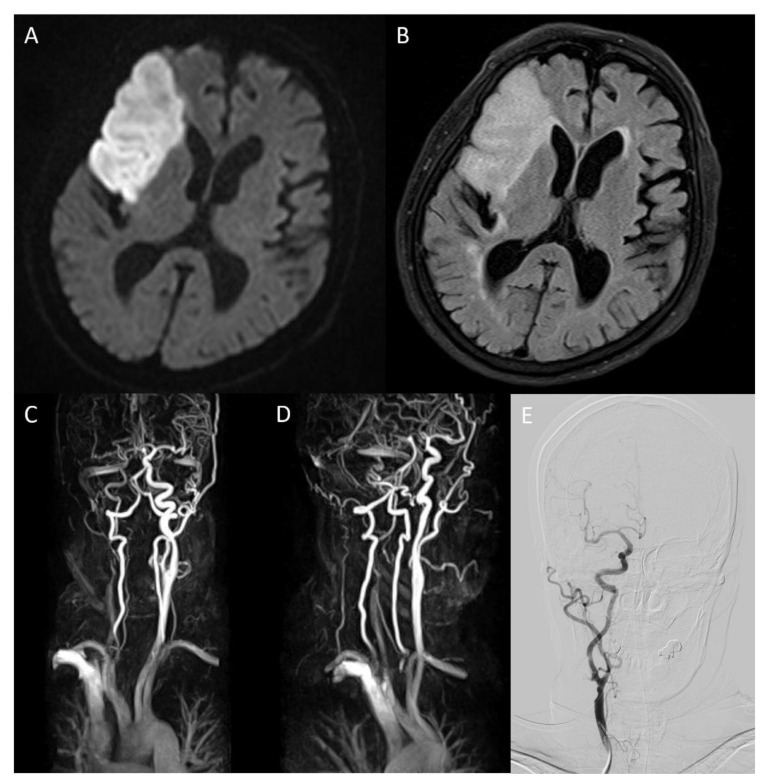
On brain magnetic resonance diffusion-weighted image (DWI) conducted after admission, infarction in middle cerebral artery territory was confirmed (**A**). On fluid-attenuated inversion recovery, high-intensity signals in the same area were detected (**B**). Magnetic resonance angiography (MRA) showed severe stenosis ranging from the right carotid bulb to the right ICA, causing decreased blood flow to the brain ((**C**), arrow). The overall state of the carotid bulb and ICA was examined through sagittal rotation of MRA (**D**). Spontaneous regression of former severe stenosis to mild degrees was confirmed on right ICA angiography of digital subtraction angiography on hospitalization day 16 (**E**).

An 86-year-old female patient visited the emergency room complaining of left hemiparesis on waking. This occurred 30 h before her hospital visit. The patient was diagnosed with hypertension 10 years ago and was on medication. She was also diagnosed with hyperlipidemia but refused to take the medication. A neurological examination was performed immediately. The patient exhibited facial palsy, left hemiparesis, and dysarthria. The National Institute of Health Stroke Scale (NIHSS) score was 4, and mental status was alert. Electrocardiography demonstrated atrial fibrillation. The chest radiography revealed cardiomegaly. Brain magnetic resonance (MR) diffusion-weighted imaging (DWI) and fluid-attenuated inversion recovery (FLAIR) detected high-intensity signals suggestive of acute infarction in the right middle cerebral artery (MCA) territory (Figure 1A,B). Severe stenosis of the right internal carotid artery (ICA) was also confirmed on MR angiography (Figure 1C). Laboratory results indicated increased LDL cholesterol (190 mg/dL), total cholesterol (277 mg/dL), and triglyceride (253 mg/dL). Lowered HDL cholesterol (36.4 mg/dL) was also shown in the results. Other laboratory results were normal. The patient was prescribed dual antiplatelet agents (aspirin and clopidogrel) and statin (rosuvastatin 20 mg/day) on admission.

**Figure 2:**
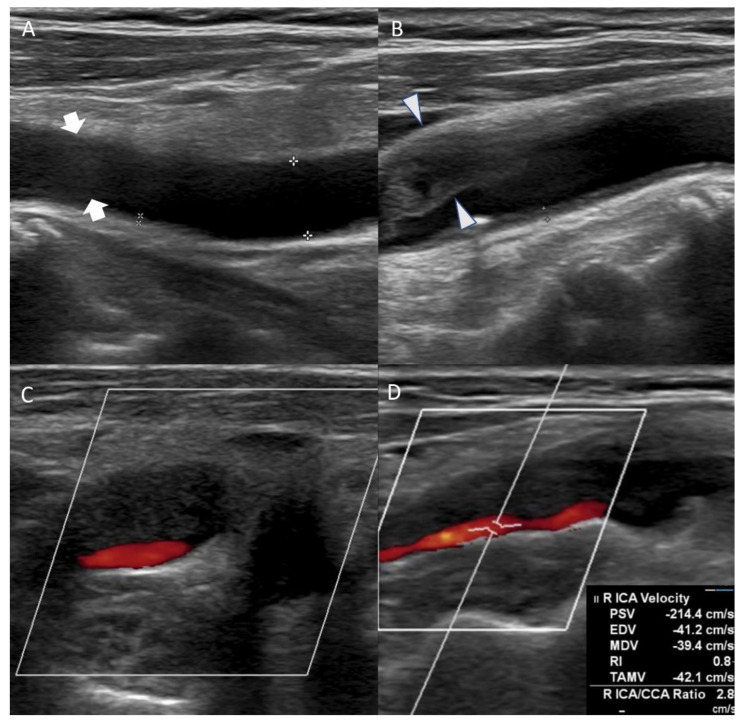
Carotid duplex ultrasonography conducted on the eleventh day of hospitalization showed echolucent (hypoechoic) plaque of right common carotid artery ((**A**), arrow) and extensive stenosis of right carotid artery bulb ((**B**), arrowhead). Axial view of the carotid artery bulb presented heterogeneous plaque suggesting lipid and calcification (**C**). Echolucent (hypoechoic) plaque that occluded right proximal internal carotid artery was observed, and peak systolic velocity of the internal carotid artery (ICA) stenosis site was 214.4 cm/s (**D**).

On the eighth day of hospitalization, the patient complained of dyspnea. Pleural effusion in the right lung was confirmed by follow-up chest X-ray, and the management for pleural effusion was initiated. On the eleventh day of hospitalization, carotid duplex ultrasonography showed extensive stenosis from the right carotid artery bulb to the proximal ICA (Figure 2). Peak systolic velocity (PSV) measured at the site of ICA stenosis was 214.4 cm/s, indicating moderate to severe grade stenosis (Figure 2). Although the patient needed (DSA) and carotid artery stenting to manage the symptomatic CAS, these procedures were delayed to the sixteenth day of hospitalization because of her aggravated general condition due to pleural effusion. On the right ICA angiogram of the DSA, the previously confirmed moderate to severe stenosis had almost regressed. Only mild stenosis with ulcerative plaque was observed (Figure 1D, 15% of the North American Symptomatic Carotid Endarterectomy Trial, NASCET). Therefore, carotid stenting was not performed, and we focused on acute stroke management. There were no additional lesions on the follow-up MRI. The patient was subsequently discharged without significant problems. One month after discharge, the patient’s lipid profile improved and the results are as follows: LDL cholesterol (116 mg/dL), total cholesterol (195 mg/dL), triglyceride (155 mg/dL), and HDL cholesterol (48 mg/dL).

## 3. Discussion

In cases of CAS > 50%, endarterectomy or stent insertion is indicated within two weeks of symptoms onset [4]. Our patient had right proximal ICA stenosis with right MCA territorial infarction and severe stenosis with an unstable lipid-rich plaque extending from the right carotid bulb to the proximal ICA on carotid duplex ultrasonography. Dual antiplatelet agents (aspirin and clopidogrel) and intensive statin therapy were started immediately after admission. Regression of the severe stenosis at the right proximal ICA was evident on DSA after two weeks of medications.

Atherosclerosis, the major cause of CAS, progresses through chronic inflammation following sub-endothelial lipid deposition in the arterial wall [8]. The most common site of carotid artery plaque is carotid artery bifurcation. Typically, there are three possible processes by which atherosclerosis induces CAS. First, with a large amount of lipid accumulation, arterial wall thickening can narrow the arterial lumen even without thrombus formation. This multilayered plaque is composed of several lipid cores, accumulated inflammatory cells, and microhemorrhages caused by neovascularization. Second, atherosclerosis causes deformity of the endothelial cell surface, resulting in blood flow changes in the arterial wall that induces thrombus formation. Third, when the unstable plaque ruptures and forms ulcerations, thrombotic occlusion may occur [2]. In our patient, ultrasonography demonstrated irregular arterial wall and plaque ulceration. Hence, thrombus after plaque rupture is the most likely cause of CAS in this case.

Recently, intensive statin therapy has been shown to have a therapeutic effect on atherosclerotic plaque regression. According to the ASTEROID trial in 2006, patients treated with intensive statin therapy for 24 months showed an average 2.47% reduction in percent atheroma volume (PAV) [6]. Moreover, in the 2019 STAMINA-MRI study, 6 months of intensive statin therapy resulted in an average decrease of 16.24% [7]. These findings have resulted from long-term intensive statin therapy. Therefore, it can be inferred that long-term intensive statin therapy is essential for a meaningful level of atherosclerotic plaque regression. In contrast, in our case, severe CAS with untreated dyslipidemia regressed rapidly. Therefore, what is the cause of rapid plaque regression in our patient, and what is the pathogenesis of atherosclerosis and its regression?

The first mechanism that may have potentially caused the patient’s rapid plaque regression is the anti-inflammatory action of M2 macrophages. In atherosclerotic plaques, M2 macrophages clear apoptotic M1 cells and necrotic debris and promote tissue repair and remodeling. As a result, the M2 macrophages stabilize the plaques and decrease plaque vulnerability. In particular, M2c macrophages, a subtype related to the anti-inflammatory function of M2 macrophages, are polarized by cytokines, such as IL-10 and transforming growth factor-β (TGF-β) [9]. According to Rodríguez-Vita et al., statins not only increase TGF- β synthesis in vascular smooth muscle cells, which play an important role in atherogenesis but also activate the TGF-β signaling pathway [10]. Therefore, two weeks of intensive statin therapy in this patient may have activated TGF-β signaling, which increased M2 macrophage polarization that exerted a protective role against atherosclerosis.

Second, reduced foam cell formation may have led to the rapid plaque regression. The statin and aspirin treatment started after admission would have negatively impacted foam cell formation. Statins reduce serum cholesterol levels by inhibiting HMG-CoA reductase in hepatocytes, which plays an essential role in cholesterol biosynthesis, and consequently prevents macrophages from ingesting LDL [11]. Furthermore, aspirin increases the expression of scavenger receptor class B type I (SR-BI) protein, which is involved in foam cell inhibition. SR-B1, a receptor protein on the cell surface, is predominantly expressed in cells such as atherosclerotic plaque macrophages that require cholesterol homeostasis, and it reduces foam cell formation by promoting cellular efflux of cholesterol [8]. Recent studies on carotid plaque specimens obtained from patients treated with aspirin support a remarkable increase in SR-BI expression [12]. In our patient, hyperlipidemia may have been normalized quickly by intensive statin therapy, and aspirin treatment may have increased SR-B1 expression, resulting in a decrease in pro-inflammatory cytokines from foam cells. As a result of these processes, atherosclerosis formation may have been inhibited.

The last mechanism to consider is the possibility of increased endogenous fibrinolysis. TNF-α, and IL-6, pro-inflammatory cytokines released by foam cells, increase the expression of plasminogen activator inhibitor (PAI-1), which inactivates tissue plasminogen activator (t-PA) [13,14]. Our patient may have a high plasma PAI-1 level because of the large number of foam cells resulting from untreated hyperlipidemia. However, aspirin and intensive statin therapy dramatically reduced foam cells. Decreased PAI-1 expression by foam cell reduction would have resulted in increased activation of endogenous t-PA. According to Sahebkar et al., statin therapy results in a reduction in plasma PAI-1 levels [15]. Our patient probably had thrombus volume reduction through the same mechanism.

There is another possibility that can be considered. Malinow suggested ‘spontaneous waxing and waning of plaque’. According to Malinow, in plaque mainly composed of fibrin-platelet thrombus, the thrombus is organized into fibrous tissue after condensation and contraction regardless of lipid control [16]. However, the patient’s total cholesterol level decreased from 277 mg/dL to 195 mg/dL. Heterogeneous plaque consisting of lipid and calcification was also observed in carotid duplex ultrasonography. Therefore, the theory is difficult to fully explain the rapid regression of atherosclerotic plaque in this case.

While the exact mechanism associated with the rapid regression of CAS remains unclear, the three aforementioned mechanisms may have led to rapid CAS regression together in our patient. In other words, the plaque volume seems to have decreased at the same time the untreated hyperlipidemia was rapidly controlled. The decrease in foam cells by statin and aspirin would have also reduced inflammation and thereby increased thrombolytic activity. Despite the use of statin, antiplatelet therapy, and even recombinant t-PA, atherosclerotic plaques in most patients show slow regression. Intensive inflammation control on atherosclerosis in a short period and the therapeutic response to statin are critical to rapid plaque regression in this patient. Further research is warranted.

Additional carotid duplex sonography was also planned to be performed after the patient’s discharge. However, the patient was admitted due to sudden hematochezia and expired during hospitalization, which leads to the limitation of this case report that comparison of the same modality before and after plaque regression was unfulfilled. Nevertheless, since the plaque was confirmed several times with various imaging methods, the results can explain the patient’s atherosclerotic plaque and its regression. Therefore, this case report is clinically meaningful in that it demonstrated a remarkable therapeutic effect of short-term intensive statin and aspirin therapy on atherosclerosis with untreated hyperlipidemia. If future studies with multiple patients will reproduce these results, the effect will be confirmed.

## Data Availability

We can provide clinical data to editors upon appropriate request.
